# Solving Traveling Salesman Problems Based on Artificial Cooperative Search Algorithm

**DOI:** 10.1155/2022/1008617

**Published:** 2022-04-12

**Authors:** Guangjun Liu, Xiaoping Xu, Feng Wang, Yangli Tang

**Affiliations:** ^1^School of Sciences, Xi'an University of Technology, Xi'an 710054, China; ^2^School of Mathematics and Statistics, Xi'an Jiaotong University, Xi'an 710049, China

## Abstract

The traveling salesman problem is a typical NP hard problem and a typical combinatorial optimization problem. Therefore, an improved artificial cooperative search algorithm is proposed to solve the traveling salesman problem. For the basic artificial collaborative search algorithm, firstly, the sigmoid function is used to construct the scale factor to enhance the global search ability of the algorithm; secondly, in the mutation stage, the DE/rand/1 mutation strategy of differential evolution algorithm is added to carry out secondary mutation to the current population, so as to improve the calculation accuracy of the algorithm and the diversity of the population. Then, in the later stage of the algorithm development, the quasi-reverse learning strategy is introduced to further improve the quality of the solution. Finally, several examples of traveling salesman problem library (TSPLIB) are solved using the improved artificial cooperative search algorithm and compared with the related algorithms. The results show that the proposed algorithm is better than the comparison algorithm in solving the travel salesman problem and has good robustness.

## 1. Introduction

Traveling salesman problem (TSP) is not only a basic circuit problem but also a typical NP hard problem and a typical combinatorial optimization problem. It is one of the most famous problems in the field of mathematics [1, 2]. It was first proposed by Menger in 1959. After it was proposed, it has attracted great attention of scholars and managers in operations research, logistics science, applied mathematics, computer application, circle theory and network analysis, combinatorial mathematics, and other disciplines and has become a research hotspot in the field of operations research and combinatorial optimization [3]. In recent years, many scholars have studied the TSP [4–9], and more scholars have expanded the traveling salesman problem [10–12]. At present, the traveling salesman problem is widely used in various practical problems such as Internet environment, road traffic, and logistics transportation [13]. Therefore, the research on TSP has important theoretical value and practical significance.

In the past 30 years, intelligent optimization algorithms have been favored by scholars because of their few parameters, simple structure, and easy implementation such as genetic algorithm (GA) [14–17], differential evolution algorithm (DE) [18], invasive weed optimization (IWO) [19], and particle swarm optimization (PSO) [20]. Artificial cooperative search algorithm was firstly proposed by Pinar Civicioglu in 2013 to solve numerical optimization problems [21]. The algorithm was proposed to simulate the interaction and cooperation process between two superorganisms with predator-prey relationship in the same natural habitat. In nature, the amount of food that can be found in an area is very sensitive to climate change. Therefore, many species in nature will migrate to find and migrate to higher yield breeding areas. It includes the processes of predator selection, prey selection, mutation, and crossover [22–25]. Firstly, the predator population location is randomly generated, the predator location memory is set, and then the prey population location is randomly generated to reorder the prey location, where biological interactions occur during the variation phase. Finally, enter the crossover stage and update the biological interaction position through the active individuals in the predator population. Compared with other optimization algorithms, artificial cooperative search algorithm has the advantages of less control parameters and strong robustness and adopts different mutation and crossover strategies. At present, the algorithm has been used to solve scheduling problems, design problems, and other practical problems [26, 27]. To some extent, these methods do solve some practical problems, but there are still some defects such as slow convergence speed and low accuracy. Therefore, it is necessary to improve artificial cooperative search algorithm to improve the performance of the algorithm [28–30].

Aiming at the disadvantages of slow convergence speed, low accuracy, and easy to fall into local optimization of the basic artificial cooperative search algorithm (ACS), this paper proposes a reverse artificial cooperative search algorithm based on sigmoid function (SQACS), that is, after constructing the scale factor by the sigmoid function, the DE/rand/1 mutation strategy of differential evolution algorithm is added in the mutation stage, and the quasi-reverse learning strategy is introduced in the later development stage of the algorithm. In the numerical simulation, the SQACS is used to solve several examples in TSPLIB. The results show that the presented algorithm is feasible.

The remainder of this paper is organized in the following manner.  Section 2 describes the TSP model. In Section 3, the basic and improved ACS algorithms are introduced in detail. Solving TSP by the SQACS is described in Section 4.  Section 5 covers simulations that have been conducted, while Section 6 presents our conclusion.

## 2. TSP Model

In general, TSP specifically refers to a traveling salesman who wants to visit *n* cities, starting from a city, must pass through all the cities only once, and then return to the departure city, requiring the traveling agent to travel the shortest total distance [13]. It is described in graph theory language as follows. In a weighted completely undirected graph, it is necessary to find a Hamilton cycle with the smallest weight. That is, let *G*=(*V*, *E*), *V*={1,2, ⋯, *n*} represent the set of vertices and *E* represent the set of edges, and each edge *e*=(*i*, *j*) ∈ *E* has a non-negative weight *m*(*e*). Now it is necessary to find the Hamilton cycle *C* of *G* so that the total weight *M*(*C*)=∑_*E*(*C*)_*m*(*e*) of *C* is the smallest. If *d*_*ij*_ is used to represent the distance between city *i* and city *j*, *d*_*ij*_ ≥ 0, *i*, *j* ∈ *v*, $x_{ij}=\left\{\begin{array}{cc} 1, & \mathrm{edge}\;\left(i,j\right)\text{ is on the optimal path}\\ 0, & \text{otherwise} \end{array}\right.$, then the mathematical model of TSP is as follows:

(1) $$\begin{array}{c} \min\;Z=\sum \sum d_{ij}x_{ij}, \end{array}$$

(2) $$\begin{array}{c} \text{s}.\text{t}.\;\sum_{j\neq 1}x_{ij}=1\;i\in V, \end{array}$$

(3) $$\begin{array}{c} \sum_{i\neq j}x_{ij}=1\;j\in V, \end{array}$$

(4) $$\begin{array}{c} \sum_{i,j\in S}x_{ij}=\left|S\right|-1\;S\subseteq V, \end{array}$$

(5) $$\begin{array}{c} x_{ij}\in \left\{0,1\right\}\;i,j\in V, \end{array}$$

where |*S*| represents the number of vertices in the set *S*. The first two constraints shown in (2) and (3) indicate that there is only one inbound and one outbound edge for each vertex, and the third constraint (4) indicates that no sub-loops will be generated.

## 3. Artificial Cooperative Search Algorithm

### 3.1. Basic Artificial Cooperative Search Algorithm

Basic artificial cooperative search (ACS) algorithm is a global search algorithm based on two populations, which is used to solve numerical optimization problems [21]. Generally, ACS includes the following population initialization, predator selection, prey selection, mutation, crossover, update selection, and so on.

#### 3.1.1. Population Initialization

ACS contains two superorganisms: *α* and *β*, in which *α* and *β* contain artificial sub-superorganisms equal to the population size (*N*). In the relevant sub-superorganisms, the number of individuals is equal to the dimension (*D*) of the problem. *α* and *β* ultrasound organisms are used to detect artificial predators and prey sub-superorganisms. The initial values of the sub-superorganism of *α* and *β* are defined by the following:

(6) $$\begin{array}{c} \alpha_{i,j}=low_{j}+R\left(0,1\right)\times \left(up_{j}-low_{j}\right), \end{array}$$

(7) $$\begin{array}{c} \beta_{i,j}={\text{low}}_{j}+R\left(0,1\right)\times \left({\text{up}}_{j}-{\text{low}}_{j}\right), \end{array}$$

where *i*=1,2, ⋯, *N*, *N* is the population size, *j*=1,2, ⋯, *D*, *D* is the dimension of the optimization problem, *α*_*i*,*j*_ and *β*_*i*,*j*_ are the components of the *i*-th sub-superorganism in the *j*-th dimension, up_*j*_ and low_*j*_ are the upper and lower limits of the *j*-th dimension search interval, respectively, and *R*(0,1) is a random number uniformly distributed on [0, 1].

#### 3.1.2. Predator Selection

At this stage of ACS, the cooperative relationship between two artificial superorganisms is defined. In each iteration of ACS, according to the “if then else” rule, the artificial predator sub-superorganism is randomly defined from two artificial superorganisms (*α* and *β*), and the artificial predator is selected through (8). At this stage of ACS, in order to help explore the search space of the problem and promote the utilization of high-quality solutions, a memory process is developed. In order to provide this memory process, during coevolution, artificial predators will follow artificial prey for a period of time to explore more fertile eating areas.

(8) $$\begin{array}{c} \text{predator}=\left\{\begin{array}{cc} \alpha ,\text{key}=1. & r_{1}<r_{2}\\ \beta ,\text{key}=2. & \text{otherwise} \end{array}\right., \end{array}$$

where *r*_1_ and *r*_2_ are uniformly distributed random numbers on the [0, 1] interval, predator represents the predator, key represents the memory that tracks the origin of the predator in each iteration, and its memory is used to improve the performance of the algorithm.

#### 3.1.3. Prey Selection

Using the same rules as selecting artificial predators, artificial prey is selected through two artificial superorganisms (*α* and *β*). In ACS, the hierarchical sequence of artificial prey is replaced by random transformation function, which is used to simulate the behavior of superorganisms living in nature. The artificial prey is selected by (9), and the selected prey is used to define the search direction of ACS in each iteration.

(9) $$\begin{array}{c} prey=\left\{\begin{array}{cc} \alpha , & r_{1}<r_{2}\\ \beta , & \text{otherwise} \end{array},\right. \end{array}$$

where *r*_1_ and *r*_2_ are uniformly distributed random numbers in the [0, 1] interval and *prey* represents prey.

#### 3.1.4. Mutation

Using the mutation process defined in equation (10), the biological interaction position between artificial predator and prey sub-superorganism is simulated. The algorithm embeds a walk process (random walk function) in the mutation process to simulate the foraging behavior of natural superorganisms. In order to promote the exploration of the problem search space and the development of more effective solutions, the variation matrix is generated by using some experience obtained by the artificial predator sub-superorganism in the previous iteration.

(10) $$\begin{array}{c} X_{i}\left(iter+1\right)={\text{predator}}_{i}\left(iter\right)+R\times \left({\text{prey}}_{i}\left(iter\right)-{\text{predator}}_{i}\left(iter\right)\right),\\ R=\left\{\begin{array}{cc} 4\times a\times \left(b-c\right) & r_{1}<r_{2}\\ \Gamma \left(4\times rand,1\right), & \text{otherwise} \end{array}\right., \end{array}$$

where in order to control the scale factor of biological interaction speed, it is calculated from (13). *iter* is the current number of iterations, *i* ∈ {1,2, ⋯, *N*}, *a*, *b*, *c*, *rand*, *r*_1_ and *r*_2_ are random numbers uniformly distributed on the [0,1] interval, and Γ is the gamma distribution with shape parameter 4 × *rand* and scale parameter 1.

#### 3.1.5. Crossover

As defined in equation (11), the active individuals in the artificial predator sub-superorganism are determined by a binary integer matrix *M*. The initial value of *M* is a matrix whose elements in row *N* and column *D* are all 1. In ACS, those individuals who can only find new biological interaction sites and can participate in migration at any time are called active individuals. The degree of cooperation between individuals in the migration process is determined by the control parameter *P*, which limits the number of active individuals produced by each artificial sub-superorganism. Then, the parameter controls the number of individuals involved in the crossover process, that is, it determines the probability of biological interaction in the crossover process. The crossover operator of ACS is given by

(11) $$\begin{array}{c} X_{i,j}\left(iter+1\right)=\left\{\begin{array}{cc} {\text{predator}}_{i,j}\left(iter\right), & M_{i,j}>0\\ X_{i,j}\left(iter\right), & \text{otherwise} \end{array}\right.,\\ {\text{Condition}}_{i,j}=\left\{\begin{array}{cc} 1, & r_{1}>P\times r_{2}\\ 0, & \text{otherwise} \end{array}\right.,\\ M_{i,j}=\left\{\begin{array}{cc} M_{i,j}, & r_{3}>P\times r_{4}\\ M_{i,j}\times {\text{Condition}}_{i,j}, & \text{otherwise} \end{array}\right., \end{array}$$

where *i* ∈ 1,2, ⋯, *N*, *j* ∈ 1,2, ⋯, *D*. predator_*i*,*j*_ represents the component of the *i-*th predator in the *j*-th dimension, and *M*_*i*,*j*_ represents the component of the *i-*th active individual of the predator in the *j*-th dimension. *r*_1_, *r*_2_, *r*_3_, and *r*_4_ represent uniformly distributed random numbers in the [0, 1] interval, and *P* represents the probability of biological interaction. Different experiments with different *P* values in the [0.05, 0.15] interval show that ACS is not sensitive to the initial value of its control parameters.

#### 3.1.6. Update Selection

The memory *key* set in the predator selection stage updates the *α* and *β* superorganisms, so as to better select predators and prey at the beginning of the next iteration, so as to strengthen the global search performance. The specific operation is shown in (12) and (13).

(12) $$\begin{array}{c} \alpha_{i}\left(iter+1\right)=\left\{\begin{array}{cc} {\text{predator}}_{i}\left(iter\right), & key=1\\ \alpha_{i}\left(iter\right), & \text{otherwise} \end{array}\right., \end{array}$$

(13) $$\begin{array}{c} \beta_{i}\left(iter+1\right)=\left\{\begin{array}{cc} {\text{predator}}_{i}\left(iter\right), & \text{key}=2\\ \beta_{i}\left(iter\right), & \text{otherwise} \end{array}\right., \end{array}$$

where *i* ∈ 1,2, ⋯, *N*, predator_*i*_ represents the *i*-th predator, and *iter* represents the current number of iterations.

### 3.2. Improved Artificial Cooperative Search Algorithm

Because ACS is not mature and perfect in theory and practice, aiming at its shortcomings such as slow convergence speed, low accuracy, and easy to fall into local optimization, a reverse artificial cooperative search algorithm based on sigmoid function (SQACS) is proposed. The specific improvement scheme is as follows.

#### 3.2.1. Constructing Scale Factor R with Sigmoid Function

In ACS, the scale factor *R* controlling the speed of biological interaction is randomly generated, which often makes the algorithm fall into local optimization, which is not conducive to the global search of the algorithm. In order to solve this problem, the following sigmoid function is introduced:

(14) $$\begin{array}{c} y=\frac{1}{1+e^{-x}}. \end{array}$$

**Figure 1:**
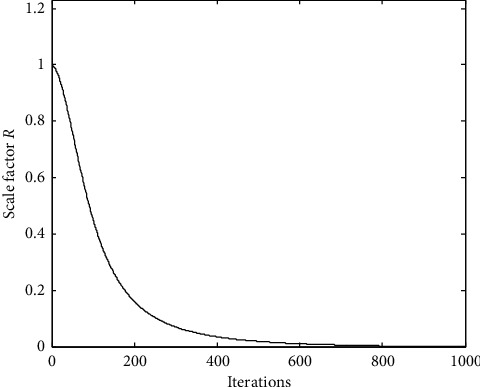
The curve of scale factor (R).

The sigmoid function is continuous, derivable, bounded, and strictly monotonic, and it is a kind of excitation function [31]. In ACS, according to the mechanism of the biological interaction position, it is known that at the beginning of the algorithm, it needs to quickly approach the optimal position. When it reaches the optimal position, it is necessary to reduce the search speed of the algorithm. Through the sigmoid function and constructing (15), the scale factor *R* that randomly controls the speed of biological interaction is transformed into a quantity that changes with the number of iterations and is mapped to the range of [0, 1], so that the scale factor *R* in [0, 1] gradually decreases in the range, so as to find the optimal solution more accurately. In this way, the scale factor *R* constructed using the sigmoid function is as in equation (15), and its curve is shown in Figure 1.

(15) $$\begin{array}{c} R\left(iter\right)=\frac{1}{1+e^{2\ln\;100\times iter/G_{\max}-\ln100}}\times \frac{G_{\max}-iter+1}{G_{\max}}, \end{array}$$

where *G*_max_ is the maximum number of iterations, *iter* is the current number of iterations, and *R*(*iter*) is the scale factor at the *iter*-th iteration.

#### 3.2.2. Quadratic Mutation Strategy

The DE/rand/1 mutation strategy of the DE is added to the second mutation of the population generated in the mutation stage of the ACS [18]. Research has found that the Gaussian, random, linear, or chaotic changes of the parameters in the DE can effectively prevent premature convergence. Therefore, after the DE/rand/1 mutation strategy of the DE is added to the ACS, a new mutation population is generated, and the next crossover behavior is performed. Thereby, the algorithm can avoid falling into the local optimum and improve the calculation accuracy. The quadratic mutation formula is

(16) $$\begin{array}{c} {\text{X}}_{i,j}\left(iter+1\right)={\text{X}}_{r1,j}\left(iter\right)+sf\times \left({\text{X}}_{r2,j}\left(iter\right)-{\text{X}}_{r3,j}\left(iter\right)\right), \end{array}$$

where *i* ∈ 1,2, ⋯, *N*, *j* ∈ 1,2, ⋯, *D*, *iter* is the current iteration number, random integers *r*1, *r*2, *r*3 ∈ *N*, and *r*1 ≠ *r*2 ≠ *r*3 ≠ *i*. The variation factor *sf* is a control parameter that scales any two of the three vectors and adds the scaled difference to the third vector. In order to avoid search stagnation, the variation factor *sf* usually takes a value in the range of [0.1, 1].

#### 3.2.3. Quasi-Reverse Learning Strategy

In the later development stage of the algorithm, a better biological interaction position should be found between the populations. Because the position is changing and this change is random, it often prevents it from searching for the optimal solution in a small local area. In order to overcome the above shortcomings, a pseudo-reverse learning strategy is introduced to generate pseudo-reverse populations to increase the diversity of the populations, so that organisms can conduct detailed search for interaction positions in neighboring communities to avoid skipping the optimal solution, and then greedy selection from the current population and quasi-reverse population can effectively find the optimal solution [32–35]. The detailed process is given below:

(i) Assuming that X=(*x*_1_, *x*_2_, ⋯, *x*_*n*_) is a *n*-dimensional solution, *x*_1_, *x*_2_, ⋯, *x*_*n*_ ∈ *R* and *x*_*i*_ ∈ [*l*_*i*_, *u*_*i*_], *i* ∈ {1,2, ⋯, *n*}. Then, the reverse solution $\text{OX}=\left({\overset{⌣}{x}}_{1},{\overset{⌣}{x}}_{2},\cdots ,{\overset{⌣}{x}}_{n}\right)$ can be defined as
  (17) $$\begin{array}{c} {\overset{⌣}{\text{x}}}_{i}=l_{i}+u_{i}-x_{i}. \end{array}$$
(ii) On the basis of the reverse solution, the quasi-reverse solution $\text{QOX}=\left({\overset{⌣}{x}}_{1}^{q},{\overset{⌣}{x}}_{2}^{q},\cdots ,{\overset{⌣}{x}}_{n}^{q}\right)$ can be defined as
  (18) $$\begin{array}{c} {\overset{⌣}{x}}_{i}^{q}=\text{rand}\left(\frac{l_{i}+u_{i}}{2},{\overset{⌣}{x}}_{i}\right). \end{array}$$

In this way, the choice of the quasi-inverse solution and the current solution is

(19) $$\begin{array}{c} X=\left\{\begin{array}{cc} {\overset{⌣}{x}}_{i}^{q}, & f\left({\overset{⌣}{x}}_{i}^{q}\right)<f\left(x_{i}\right)\\ x_{i}, & \text{otherwise} \end{array}\right.. \end{array}$$

**Figure 2:**
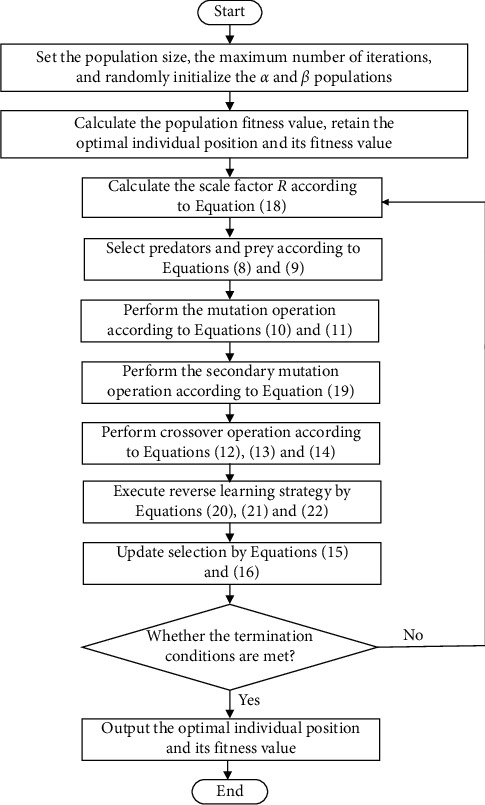
Flowchart of SQACS.

To sum up, the flowchart of the proposed SQACS is shown in Figure 2.

## 4. Solving TSP by SQACS

Taking the shortest path (i.e., equation (1)) as the objective function, the SQACS is used to solve the TSP. In order to better solve the TSP and realize the transformation between biological organism and TSP solution space, each biological interaction location X_*i*_=(*x*_*i*1_, *x*_*i*2_, ⋯, *x*_*in*_) is defined as a sequence of traversing and accessing each city number in this paper. For example, one of the interaction positions X_*i*_=[1,3,2,4,5,6] means that the TSP is that the traveler first visits the city with number 1, then successively visits the cities with numbers 3, 2, 4, 5 and 6, and finally returns to the departure city, that is, the city with number 1, and the corresponding objective function is equivalent to the path length of TSP. For the TSP, the shorter the individual's visit path is, the greater the fitness value is, so the fitness function *f*(*x*_*i*_)=1/*Z*_*x*_*i*__ is selected, where *i*=1,2, ⋯, *n*, *n* is the number of cities to visit, and the lower and upper limits of variables are 1 and *n*, respectively. The specific steps of SQACS to solve TSP are as follows:

 
*Step 1*. Population initialization: use the city number to encode the TSP path and randomly generate the arrangement order of *n* cities.
 
*Step 2*. Calculate the fitness value of each individual in the population.
 
*Step 3*. Randomly select the predator and prey population, and then randomly rearrange the position of prey population.
 
*Step 4*. Calculate the scale factor *R* of biological interaction velocity.
 
*Step 5*. Determination of active individuals *M* in predator population by binary integer mapping.
 
*Step 6*. Mutation: calculate the location *X* of biological interaction, i.e., the visit route of the traveler.
 
*Step 7*. Crossover: if the active individual mapping is greater than 0, update the path to the predator location; otherwise, keep the original location unchanged.
 
*Step 8*. Reselection of predator and prey populations.
 
*Step 9*. Judge whether the termination conditions are met. If so, stop the algorithm update and output the optimal position and optimal function value, that is, the shortest route and shortest path value of TSP. Otherwise, return to Step 2.

## 5. Numerical Simulation

In order to verify the performance of the proposed SQACS, SQACS is tested with GA [14], DE [18], IWO [19], PSO [20], ACS [21], IACS1 [27], and IACS2 [36] to solve four TSPs of different scales in TSPLIB standard database: Oliver 30, Att48, Eil51, and Eil76. In the simulation, in order to compare the results under the same conditions as much as possible, the maximum evaluation times of each algorithm is 2000 and the initial population size is 20. Other parameter settings of the algorithm involved are shown in the corresponding references.

After 30 times of solution by SQACS and the above-mentioned algorithms, the optimal value, average value, and average calculation time of the results are shown in Table 1. By comparing the optimal value and average value of each algorithm to solve the TSP, it can be seen that SQACS can obtain the optimal results in the minimum value and average value, and the difference between the optimal value and average value is the smallest, indicating that SQACS has the strongest stability. From the comparison of index values of calculation time, SQACS performs better than other comparison algorithms on the four datasets. It can be seen that SQACS has good feasibility and robustness in solving TSP.

**Figure 3:**
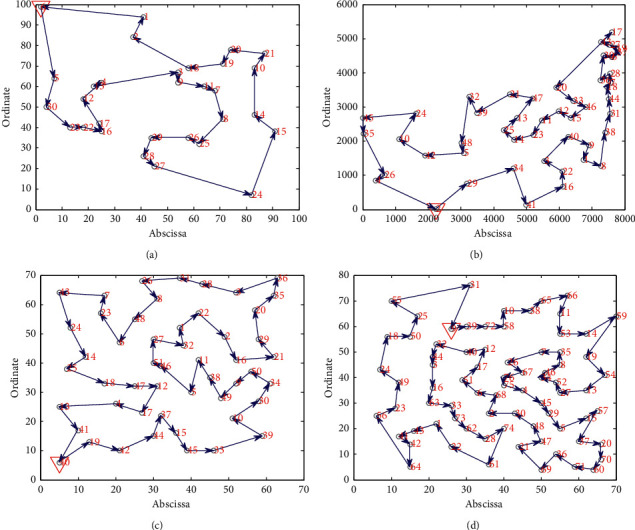
The optimal path diagrams of four examples obtained by the SQACS. (a) Oliver30. (b) Att48. (c) Eil51. (d) Eil76.

Figure 3 shows the optimal path diagram of four groups of examples in solving TSP by SQACS. As can be seen from Figure 3, except the path intersection in Att48 dataset, the other three figures are a completely closed loop, and the paths do not cross, so the obtained paths are feasible. Also, the solutions of the optimal route obtained are as follows: 6⟶5⟶30⟶23⟶22⟶16⟶17⟶12⟶13⟶4⟶3⟶9⟶11⟶7⟶8⟶25⟶26⟶29⟶28⟶27⟶24⟶15⟶14⟶10⟶21⟶20⟶ 19⟶18⟶2⟶1⟶6; 2⟶29⟶34⟶41⟶16⟶22⟶3⟶40⟶9⟶1⟶8⟶38⟶31⟶44⟶18⟶7⟶28⟶36⟶30⟶6⟶37⟶19⟶27⟶17⟶43⟶20⟶ 33⟶46⟶15⟶12⟶11⟶23⟶14⟶25⟶13⟶47⟶24⟶39⟶32⟶48⟶5⟶42⟶10⟶24⟶45⟶35⟶26⟶4⟶2; 40⟶19⟶42⟶44⟶37⟶15⟶45⟶33⟶39⟶10⟶30⟶34⟶50⟶9⟶49⟶38⟶11⟶5⟶46⟶51⟶27⟶32⟶1⟶22⟶2⟶16⟶21⟶29⟶20⟶35⟶36⟶3⟶28⟶31⟶26⟶8⟶48⟶6⟶23⟶7⟶43⟶24⟶14⟶25⟶18⟶47⟶12⟶17⟶4⟶13⟶41⟶40; 9⟶39⟶72⟶58⟶10⟶38⟶65⟶56⟶11⟶53⟶14⟶59⟶19⟶54⟶13⟶27⟶52⟶34⟶46⟶8⟶35⟶7⟶26⟶67⟶76⟶ 75⟶4⟶45⟶29⟶5⟶15⟶57⟶37⟶20⟶70⟶60⟶74⟶36⟶ 69⟶21⟶47⟶48⟶30⟶2⟶68⟶6⟶51⟶17⟶12⟶40⟶32⟶ 44⟶3⟶16⟶63⟶33⟶73⟶62⟶28⟶74⟶61⟶22⟶1⟶43⟶ 41⟶42⟶64⟶56⟶23⟶49⟶24⟶18⟶50⟶25⟶55⟶31⟶9.

In order to further verify the effectiveness of SQACS, the algorithms in [5–9] are further selected to compare the solution results of Oliver 30, Att48, Eil51, and Eil76 in TSP. The comparison results are shown in Table 2. By comparing the data in Table 2, it can be found that the solution results of SQACS on the other three datasets are better than those proposed in the literature, and the solution results all reach the optimal value in TSPLIB database. This verifies the effectiveness of SQACS in solving TSP.

## 6. Conclusion

In order to better solve the traveling salesman problem, this paper proposes a reverse artificial collaborative search algorithm based on sigmoid function. That is, the scale factor is constructed by sigmoid function to improve the global search ability of the algorithm. In the mutation stage, the DE/rand/1 mutation strategy of differential evolution algorithm is added to carry out secondary mutation on the current population, so that the algorithm can avoid falling into local optimization and improve the calculation accuracy. In the later development stage of the algorithm, the quasi-reverse learning strategy is introduced to find the optimal solution more effectively. Finally, the proposed algorithm is used to solve the traveling salesman problem, and the results show that the proposed algorithm in this paper is effective for solving the traveling salesman problem.

## Figures and Tables

**Table 1:** Comparison of experimental results of eight algorithms.

Example	Evaluation criterion	GA	DE	IWO	PSO	ACS	IACS1	IACS2	SQACS
Oliver30	Optimal value	427.37	423.11	431.34	428.01	453.27	452.12	434.67	420.00
	Average value	432.33	435.94	449.98	437.83	475.23	468.21	440.62	421.31
	Average time/s	28.36	28.28	29.67	27.26	27.41	27.85	28.26	26.17
Att48	Optimal value	33942.47	33793.06	33596.81	33642.34	34663.41	34527.23	33742.84	33516.02
	Average value	40374.28	34183.58	33637.86	33785.12	35721.39	35123.31	34081.82	33583.17
	Average time/s	49.36	49.42	50.21	49.23	50.26	50.85	51.97	49.02
Eil51	Optimal value	473.56	436.81	471.36	432.99	484.05	478.67	442.43	426.00
	Average value	481.52	451.08	482.90	448.60	496.55	480.89	449.76	427.71
	Average time/s	55.54	55.35	58.36	54.65	57.25	57.78	58.02	55.27
Eil76	Optimal value	568.47	547.13	562.20	541.91	572.93	568.37	543.00	538.00
	Average value	584.01	583.11	578.37	550.68	586.33	581.81	552.45	543.79
	Average time/s	85.49	85.28	86.23	85.54	86.23	86.54	87.15	85.16

**Table 2:** Comparison of SQACS calculation results and literature.

TSP test set	TSPLIB optimal solution	SQACS optimal solution	Reference [5] optimal solution	Reference [6] optimal solution	Reference [7] optimal solution	Reference [8] optimal solution	Reference [9] optimal solution
Oliver30	420.00	420.00	—	420.00	420.00	423.74	423.74
Att48	33503.00	33516.00	36441.00	—	33522.00	—	—
Eil51	426.00	426.00	479.00	428.87	428.00	814.53	426.00
Eil76	538.00	538.00	—	544.37	547.00	—	538.00

## Data Availability

The data used and/or analyzed during the current study are available from the corresponding author on reasonable request.
